# Predictions of the accuracy of genomic prediction: connecting R^2^, selection index theory, and Fisher information

**DOI:** 10.1186/s12711-022-00700-2

**Published:** 2022-02-14

**Authors:** Piter Bijma, Jack C. M. Dekkers

**Affiliations:** 1 Animal Breeding and Genomics, Animal Sciences Group, Wageningen University and Research, Wageningen, The Netherlands; 2 Department of Animal Science, Iowa State University, Ames, IA USA

## Abstract

**Background:**

Deterministic predictions of the accuracy of genomic estimated breeding values (GEBV) when combining information sources have been developed based on selection index theory (SIT) and on Fisher information (FI). These two approaches have resulted in slightly different results when considering the combination of pedigree and genomic information. Here, we clarify this apparent contradiction, both for the combination of pedigree and genomic information and for the combination of subpopulations into a joint reference population.

**Results:**

First, we show that existing expressions for the squared accuracy of GEBV can be understood as a proportion of the variance explained. Next, we show that the apparent discrepancy that has been observed between accuracies based on SIT vs. FI originated from two sources. First, the FI referred to the genetic component that is captured by the marker genotypes, rather than the full genetic component. Second, the common SIT-based derivations did not account for the increase in the accuracy of GEBV due to a reduction of the residual variance when combining information sources. The SIT and FI approaches are equivalent when these sources are accounted for.

**Conclusions:**

The squared accuracy of GEBV can be understood as a proportion of the variance explained. The SIT and FI approaches for combining information for GEBV are equivalent and provide identical accuracies when the underlying assumptions are equivalent.

## Background

The accuracy of estimated breeding values (EBV) is a key determinant of response to selection in livestock and plant genetic improvement. For this reason, a priori prediction of the accuracy of EBV is important for the optimization of genetic improvement programs. In genomic prediction (GP), the accuracy of EBV can be increased by combining information sources, such as pedigree and marker information [1], or information from multiple genomic reference populations [2]. Hence, to understand and optimize genomic selection programs, we need to understand the effect of combining information sources on the accuracy of genomic EBV.

Dekkers et al. [3] derived predictions of the accuracy of genomic EBV (GEBV) by combining pedigree and genomic information using two approaches: a derivation based on selection index theory (SIT) vs. a derivation based on Fisher information (FI). Both approaches are based on the assumption that sampling errors, which are inherent to the pedigree-based and genomic information, are independent of each other. Nevertheless, the two approaches resulted in slightly different accuracies of GEBV. van den Berg et al. [2] used FI to predict the accuracy of GEBV when combining information from two subpopulations.

The purpose of this paper is to clarify the apparent contradiction observed by Dekkers et al. [3] between predicted accuracies based on SIT vs. FI, and to show that these two approaches are equivalent when the same assumptions are made. We will consider two common cases where information sources are combined for GP: (i) the combination of pedigree and genomic information, as in Dekkers et al. [3], and (ii) the combination of information from two subpopulations, as in van den Berg et al. [2]. We will show that the difference between the SIT and FI approaches observed by Dekkers et al. [3] originated from two sources, which, when accounted for, make the SIT and FI approaches equivalent. First, the FI referred to the genetic component that is captured by the markers, rather than the full genetic component. Second, the SIT-based derivation of the accuracy did not account for the additional increase in the accuracy of GEBV that results from a reduction of the residual variance when combining information sources.

To explain these differences, first we show that existing expressions for the squared accuracy, or reliability, of GEBV [3–5] can be understood as a proportion of the variance explained ($R^{2}$), which simplifies subsequent derivations. Next, we consider derivations for the accuracy of GEBV when combining information sources based on SIT or on FI, first for the combination of information from two subpopulations, and second for the combination of genomic and pedigree data. Throughout this manuscript, we assume that the trait follows the additive infinitesimal genetic model [6].

## Accuracy of GEBV interpreted as an ${\varvec{R}}^{2}$

In general, the reliability ($r^{2}$, i.e., squared accuracy) of best linear unbiased predictions (BLUP) of breeding values that are based on a “single” source of information (e.g., a single phenotype or an average) is equal to the proportion of variance ($R^{2}$) in the information source that is explained by the genetic effect of interest. In other words, $r^{2}$ is equal to $R^{2}$. For example, the reliability of the EBV of a sire based on a progeny test, where each offspring has a unique dam (as in cattle), is equal to the proportion of variance in the progeny means that is explained by the effect of the sire, i.e*.*:

$$r^{2} = \frac{{\sigma_{s}^{2} }}{{\sigma_{s}^{2} + \left( {\sigma_{P}^{2} - \sigma_{s}^{2} } \right)/n}},$$

where $\sigma_{P}^{2}$ is the phenotypic variance, $\sigma_{s}^{2}$ is the variance in progeny means that is explained by the effect of the sire, $n$ is the progeny group size, and $\left( {\sigma_{P}^{2} - \sigma_{s}^{2} } \right)/n$ is the residual variance of the progeny means after accounting for the sire effect. Thus, $r^{2}$ is the ratio of the variance in the progeny means that is explained by the sire over the full variance in the progeny means, which is the $R^{2}$ due to the sire. This result is equivalent to well-known expressions for the accuracy of progeny testing e.g., [7], as evident from substituting $\sigma_{s}^{2} = \frac{1}{4}h^{2} \sigma_{p}^{2}$, which yields the well-known result $r^{2} = nh^{2} /\left[ {nh^{2} + \left( {4 - h^{2} } \right)} \right]$, where $h^{2}$ is the heritability.

The reliability of GEBV follows by analogy. In the following, without loss of generality, we assume that $\sigma_{P}^{2} = 1$, so that additive genetic variance is equal to $h^{2}$. Derivations of the accuracy of GEBV make use of the concept of effective chromosomal segments [8]. By definition, effective chromosomal segments are independent, have equal variance, and together explain the full additive genetic variance [8]. For this reason, the reliability of the full GEBV is identical to the reliability of the prediction of the effect of a single segment ([5]; note that here we ignore that all markers are fitted simultaneously in GP, which will be addressed below). Moreover, with a total of $M_{e}$ effective segments that together explain the full additive genetic variance, each segment explains an amount $h^{2} /M_{e}$ of the phenotypic variance. Then, for a reference population of $N$ genotyped and phenotyped individuals, the residual variance of the mean phenotype of the $N$ individuals, after accounting for the effect of the focal segment, equals $\left( {1 - h^{2} /M_{e} } \right)/N$. In this expression, $1$ represents the phenotypic variance, $h^{2} /M_{e}$ the variance due to the focal segment, and we divide by $N$ because we consider the variance of an average of $N$ independent residuals. Hence, analogous to the derivation of the reliability of EBV based on a progeny test, the reliability of the GEBV can be found as the $R^{2}$ due to a single segment:

1a $$r^{2} = \frac{{h^{2} /M_{e} }}{{h^{2} /M_{e} + \left( {1 - h^{2} /M_{e} } \right)/N}} = \frac{{Nh^{2} /M_{e} }}{{Nh^{2} /M_{e} + 1 - h^{2} /M_{e} }}.$$

In the second term of this expression, the numerator represents the contribution of the focal segment to the variance of the mean phenotype of the $N$ individuals, while the denominator represents the full variance of this mean. If we assume that a single segment explains a negligible proportion of the phenotypic variance, such that $h^{2} /M_{e} \ll 1$, we find:

1b $$r^{2} \approx \frac{{Nh^{2} /M_{e} }}{{Nh^{2} /M_{e} + 1}}.$$

This result was first derived by Daetwyler et al. [4] (see also Appendix A in Wientjes et al*.* [5]).

Equations (1a) and (1b) ignore that the genotyped markers may capture only a proportion $q^{2}$ of the full additive genetic variance [4, 9], which has two consequences. First, since markers now explain only a proportion $q^{2} h^{2}$ of the phenotypic variance, the heritability of the component captured by markers is reduced to $q^{2} h^{2}$. Second, since genomic information predicts only the component that is captured by markers, $g_{M}$, rather than the full genetic effect, $g_{G}$, the reliability of the marker-captured component, say $r_{M}^{2}$, must be multiplied by a factor $q^{2}$ to obtain the reliability of the prediction of $g_{G}$. In other words, $r^{2} = q^{2} r_{M}^{2}$. Hence, to account for the fact that the markers capture only a proportion $q^{2}$ of the total genetic variance, we have to substitute the $h^{2}$ in Eq. (1a) by $q^{2} h^{2}$ and multiply the full equation by a factor $q^{2}$. We then obtain:

2a $$r^{2} = q^{2} r_{M}^{2} = q^{2} \frac{{\frac{{Nq^{2} h^{2} }}{{M_{e} }}}}{{\frac{{Nq^{2} h^{2} }}{{M_{e} }} + 1 - \frac{{q^{2} h^{2} }}{{M_{e} }}}} = q^{2} \frac{{\theta_{M} }}{{ \theta_{M} + 1 - q^{2} h^{2} /M_{e} }},$$

where

2b $$\theta_{M} = Nq^{2} h^{2} /M_{e} .$$

Assuming $q^{2} h^{2} /M_{e} \ll 1$, we find:

2c $$r^{2} \approx q^{2} \frac{{Nq^{2} h^{2} /M_{e} }}{{Nq^{2} h^{2} /M_{e} + 1}} = q^{2} \frac{{\theta_{M} }}{{\theta_{M} + 1 }}$$

Thus, in contrast to Eqs. (1a) and (1b), Eqs. (2a) to (2c) take into account that the markers may not capture the full genetic variance, i.e., that $q^{2} < 1$.

Both Eqs. (1a) and (1b) and Eqs. (2a) and (2c) ignore that we fit all markers simultaneously in GP, because their derivations consider a single segment at a time, disregarding the effect of also fitting the other segments. Fitting all markers simultaneously reduces the residual variance and, therefore, increases the reliability (Appendix S1 in [4]). To derive the reliability while accounting for the fitting of all segments, we can still use the $$R^{2}$$ due to a single segment, but we have to remove the variance that is explained by the estimates of the $$M_{e} - 1$$ other segments from the residual variance, which is equal to $$\left( {M_{e} - 1} \right) r^{2} h^{2} /M_{e}$$, where $$h^{2} /M_{e}$$ is the variance of the true effects of the segments, which is multiplied by $$r^{2}$$ because we remove the variance of the estimated effects of those segments. Subtracting $$\left( {M_{e} - 1} \right) r^{2} h^{2} /M_{e}$$ from the ($$1 - q^{2} h^{2} /M_{e} )$$ term in the denominator of the second term in Eq. (2a), results in the following residual variance [2, 4, 9]:

$$1 - q^{2} h^{2} /M_{e} - \left( {M_{e} - 1} \right)r^{2} h^{2} /M_{e} = 1 - h^{2} (q^{2} - r^{2} + r^{2} M_{e} )/M_{e} ,$$

where, the first term on the left-hand side is the phenotypic variance, the second term is the variance of the true effect of the focal segment, and the third term is the variance of the estimated effects of the remaining $\left( {M_{e} - 1} \right)$ segments. Assuming $h^{2} \left( {q^{2} - r^{2} } \right)/M_{e} \ll 1$, the residual variance can be approximated by $1 - r^{2} h^{2}$. Hence, from Eq. (2a), we find:

3a $$r^{2} \approx q^{2} \frac{{Nq^{2} h^{2} /M_{e} }}{{Nq^{2} h^{2} /M_{e} + 1 - r^{2} h^{2} }} = q^{2} \frac{{\theta_{M} }}{{\theta_{M} + 1 - r^{2} h^{2} }}.$$

Note that $r^{2}$ appears on both sides of the equal signs in Eq. (3a), resulting in a quadratic equation in $r^{2}$.

Equation (3a) is identical to Eq. 1 of Dekkers et al. [3], except for $r^{2} h^{2}$ in the denominator instead of $r^{2} q^{2} h^{2}$. Our derivation results in $r^{2} h^{2}$, because the proportion of phenotypic variance that is explained by the estimated effects of all segments equals $r^{2} h^{2}$, not $r^{2} q^{2} h^{2}$. Equation (3a) is also equal to Eq. 1 of van den Berg et al. [2] when $q^{2} = 1$. While we have obtained Eq. (3a) here as the $R^{2}$ of a single segment, a derivation based on SIT yields the same result (see Appendix 10).

To find $r^{2}$, we have to solve the quadratic Eq. (3a), which yields two solutions, one of which is greater than 1 and thus irrelevant. The relevant solution is:

3b $$r^{2} = \frac{{1 + \theta_{M} - \sqrt {\left( {1 + \theta_{M} } \right)^{2} - 4h^{2} q^{2} \theta_{M} } }}{{2h^{2} }}.$$

Equation (3b) accounts both for $q^{2} < 1$ and for the reduction of residual variance because all markers are fitted simultaneously in GP. This result is similar to Eq. 6 of Dekkers et al. [3], which is $r^{2} = \left[ {1 + \theta_{M} - \sqrt {\left( {1 + \theta_{M} } \right)^{2} - 4h^{2} q^{4} \theta_{M} } } \right]/2q^{2} h^{2}$, but accounts for having $r^{2} h^{2}$ in the denominator of Eq. (3a) vs. $r^{2} q^{2} h^{2}$ in the denominator of Eq. 1 of Dekkers et al. [3]. Note that the impact of this correction will be limited, because $q^{2}$ is typically close to 1.

## Fisher information versus selection index theory when merging information

FI is a measure of the amount of information that a data point carries about an unknown parameter. Formally, it is the variance of the score function, which then equals the expected information [10]. In this section, we use the general relationship between reliability and FI ($\theta$), as given by van den Berg et al. [2], i.e.,

4 $$r^{2} = \frac{\theta }{{\theta + 1 - r^{2} h^{2} }},$$

to connect expressions for the reliability of GEBV that are based on FI to the corresponding expressions based on SIT. First, we consider the case of merging genomic information from two subpopulations into a single reference population, followed by the merging of pedigree and genomic information, as in Dekkers et al. [3].

In the following, it is essential to realize that, in Eq. (4), $r^{2}$ and the $\theta$ must refer to the same unknown genetic effect. In other words, if we aim to find $r^{2}$ for the full genetic effect, $g_{G}$, then we have to use FI for the full genetic effect in Eq. (4). However, $\theta_{M}$ defined in Eq. (2b) and used in Eqs. (2a) and (2c) and in Eqs. (3a) and (3b) represents FI for the genetic component that is captured by markers, $g_{M}$, rather than FI for the full genetic effect, $g_{G}$. This is evident from comparing Eq. (3a) to Eq. (4). Equation (3a) can be interpreted as $r^{2} = q^{2} r_{M}^{2}$, where the term $\theta_{M} /\left( {\theta_{M} + 1 - r^{2} h^{2} } \right)$ on the right-hand side of Eq. (3a) represents $r_{M}^{2}$. Note that this term is like Eq. (4), but refers to $r_{M}^{2}$ rather than $r^{2}$. This indicates that $\theta_{M}$ in Eq. (3a) represents FI for $g_{M}$ rather than $g_{G}$. For this reason, substitution of the $\theta_{M}$ defined in Eq. (2b) into Eq. (4) yields a prediction of $r_{M}^{2}$, which is why Eq. (3a) has an additional factor $q^{2}$ to translate $r_{M}^{2}$ into $r^{2}$. The same interpretation is suggested by Eq. (2b), where $\theta_{M}$ is the proportion of phenotypic variance (i.e., $R^{2}$) that is captured by a single segment, i.e., $q^{2} h^{2} /M_{e}$, multiplied by the number of observations, $N$, which makes intuitive sense as a measure of information for $g_{M}$, rather than for $g_{G}$. Therefore, Eq. (4) can be used to predict either $r_{M}^{2}$ or $r^{2} .$ A prediction of $r_{M}^{2}$ is obtained when using $\theta_{M}$ defined in Eq. (2b) into Eq. (4). A prediction of $r^{2}$ is obtained when using $\theta$ for the full genetic effect in Eq. (4), but this requires having a value for $\theta$.

### Merging subpopulations into a single a reference population using the FI and SIT approaches

Consider a reference population of size $N$, split into two non-overlapping subpopulations of sizes $N_{1}$ and $N_{2}$, with $N = N_{1} + N_{2}$. Thus, the two subpopulations contain distinct individuals, such that the $E$ terms in $P = g_{G} + E$ are independent between subpopulations. Hence, the two subpopulations have independent sampling errors, which allows FI of the two subpopulations to be summed to obtain FI of the full reference population, as in van den Berg et al. [2]. Note, however, that independence of sampling errors does not require the individuals from one subpopulation to be genetically unrelated to individuals from the other subpopulation.

First, we ignore the reduction in residual variance that results from fitting all markers simultaneously and from joint analysis of the two populations, in order to mathematically demonstrate the equivalence of the SIT and FI approaches for this case. Realizing that $r^{2} = q^{2} r_{M}^{2}$, where $r_{M}^{2}$ follows from substituting $\theta_{M}$ defined in Eq. (2b) into Eq. (4), it follows that the reliability of GEBV based on analysis of a single subpopulation, $i$, equals:

5 $$r_{i}^{2} = q^{2} r_{M,i}^{2} = q^{2} \frac{{\theta_{M,i} }}{{\theta_{M,i} + 1 - q^{2} h^{2} /M_{e} }},$$

where $\theta_{M,i} = N_{i} q^{2} h^{2} /M_{e}$, and $i =$ 1 or 2. Because we ignore the reduction in residual variance here, we use $q^{2} h^{2} /M_{e}$ rather than $r^{2} h^{2}$ in the denominator, as explained above for Eq. (2a). In Eq. (5), only $N$ has subscript $i$ (and therefore also $\theta_{M,i}$ has subscript $i$, since it is a function of $N_{i}$), because we consider the subpopulations to be from the same overall population, such that $q^{2}$, $h^{2}$, and $M_{e}$ are identical for the two subpopulations.

A prediction of the accuracy from joint analysis of the two subpopulations using the FI approach follows from summing the FI for each subpopulation. The FI for each subpopulation follows from solving Eq. (5) for $\theta_{M,i}$, which yields:

6 $$\theta_{M,i} = \frac{{r_{i}^{2} \left( {1 - q^{2} h^{2} /M_{e} } \right)}}{{q^{2} - r_{i}^{2} }}.$$

In statistical theory, FI contributed by different information sources can be summed if the sampling errors of the information sources are independent, such that $\theta_{M} = \theta_{M,1} + \theta_{M,2}$ [10]. Hence, we can find the reliability for the combined reference population by replacing $\theta_{M,i}$ in Eq. (5) by $\theta_{M} = \theta_{M,1} + \theta_{M,2}$, giving:

$$r^{2} = q^{2} r_{M}^{2} = q^{2} \frac{{\theta_{M,1} + \theta_{M,2} }}{{\theta_{M,1} + \theta_{M,2} + 1 - q^{2} h^{2} /M_{E} }}.$$

Substituting Eq. (6) for both $\theta_{M,1}$ and $\theta_{M,2}$ and simplifying the result yields a FI-based prediction of the reliability of GEBV based on the full reference population (see Appendix 13) that is equal to:

7 $$r^{2} = \frac{{r_{1}^{2} + r_{2}^{2} - 2r_{1}^{2} r_{2}^{2} /q^{2} }}{{1 - r_{1}^{2} r_{2}^{2} /q^{4} }}.$$

Alternatively, we can derive $r^{2}$ based on SIT. The detailed derivation is given in Appendix 15 and yields exactly the same result as Eq. (7). Thus, the SIT and FI approaches yield the same predictions of the accuracy of GEBV when the additional reduction in residual variance that results from fitting all markers simultaneously and from merging the two subpopulations is ignored. Note that Eq. (7) is different from the SIT result of combining pedigree and genomic information derived by Dekkers et al. [3], (see their Eq. 8), because we consider combining genomic information from merging subpopulations.

Second, we account for the reductions in residual variance due to the merger of subpopulations into a joint reference population and due to fitting all markers simultaneously. Accounting for the effect of fitting all markers simultaneously in the SIT approach can be accommodated by including the effect of the other $M_{e} - 1$ segments as an information source in the index, as illustrated in Appendix 10, and gives identical accuracy predictions as accounting for this effect in the FI approach. However, this is complex when also considering the merger of two subpopulations into a single reference population. To avoid this complexity, we use a numerical example instead. This example will also illustrate that the difference between accuracy predictions based on the SIT approach used in Dekkers et al. [3] and resulting in Eq. (7), versus predictions based on FI result from the reduction in the residual variance when the reference population is increased. The standard SIT approach (Eq. (7) as derived from SIT in Appendix 15, and [3, 11]) ignores this reduction in residual variance, while Eqs. (3a) and (3b) account for it when we add the FI for the markers (i.e., $\theta_{M}$) of the two subpopulations.

#### Example of the impact of the reduction in residual variance when combining subpopulations

Consider two non-overlapping subpopulations of the same size, with $N_{1} = N_{2} =$ 1000, $N =$ 2000, $h^{2}$ = 0.3, $q^{2}$ = 0.8, and $M_{e}$ = 400, such that $\theta_{M,1} = \theta_{M,2}$ = 0.75 based on Eq. (2b). We choose identical subpopulation sizes because it allows us to easily illustrate the impact of the reduction in residual variance. The reliability of GEBV based on one of the two subpopulations, accounting for the reduction in residual variance from fitting all markers simultaneously, follows from Eq. (3b), giving $r^{2}$ = 0.3658. This is the reliability of GEBV for each of the two subpopulations, using only information from the respective subpopulation. Next we consider the reliability of GEBV when merging the two subpopulations. When ignoring the additional reduction in residual variance that occurs when merging the two subpopulations, the reliability based on the merged population follows from Eq. (3a) using $\theta_{M} = \theta_{M,1} + \theta_{M,2}$ = 1.5 and $r^{2}$ = 0.3658, and yields $r^{2}$ = 0.5020. The use of the original $r^{2}$ (0.3658) in Eq. (3a) means we ignore the additional reduction of the residual variance due to the increased size of the reference population. Exactly the same result is found with the SIT approach, using Eq. (7), with $r_{1}^{2} = r_{2}^{2} = 0.3658$. This result illustrates that the SIT and FI approaches yield the same reliability of predictions when the same assumptions are made. However, the full increase in accuracy from merging the two subpopulations when also accounting for the additional reduction in residual variance follows from Eq. (3b) with $\theta_{M}$ = 1.5, which yields $r^{2}$ = 0.5114. This prediction is slightly larger than the 0.5020 because of the additional reduction in residual variance when the two subpopulations are merged, which is not accounted for in common SIT approaches, such as in Dekkers et al*.* [3], Harris and Johnson [11], and Eq. (7). In principle, this reduction in residual variance can be accounted for in a SIT-based derivation by extending the pseudo-BLUP derivation of Appendix 10, which yields the identical result as the FI-based approach ($r^{2}$ = 0.5114 here) (derivations not shown due to their complexity).

### Merging pedigree and genomic information using the FI and SIT approaches

Next, we consider the combination of pedigree and genomic information for GP, as in Dekkers et al*.* [3]. Suppose we have a pedigree-based EBV, $\hat{g}_{A}$, with reliability $r_{A}^{2}$, and an EBV based on deviations of genomic relationships from pedigree relationships, $\hat{g}_{D}$, with reliability $r_{D}^{2}$, as in Dekkers et al. [3]. We assume that distinct phenotypes are used for the prediction of $\hat{g}_{A}$ and $\hat{g}_{D}$, such that the sampling errors of $\hat{g}_{A}$ and $\hat{g}_{D}$ are independent [3]. Using SIT, the reliability of the total GEBV of $g_{G}$ follows from Eq. 8 of Dekkers et al. [3]:

8 $$r_{G}^{2} = \frac{{r_{A}^{2} + r_{D}^{2} - 2r_{A}^{2} r_{D}^{2} }}{{1 - r_{A}^{2} r_{D}^{2} }}.$$

This result ignores a potential increase in the reliability that would result if combining pedigree and genomic information in a single GP analysis leads to a reduction of the residual variance (proof that this occurs is not straightforward and not given).

To derive the corresponding result based on FI, it is essential to distinguish between FI for $g_{M}$ and FI for $g_{G}$. The pedigree-based EBV relates to FI for $g_{G}$, because pedigree information captures the full genetic effect. The EBV based on deviations of genomic relationships from pedigree relationships, in contrast, relates to $g_{M}$ and $\theta_{M}$. Because the $\theta_{D}$ presented in Dekkers et al. [3] relates to $g_{M}$, while $\theta_{A}$ relates to $g_{G}$, we cannot simply add $\theta_{D}$ and $\theta_{A}$ to obtain the full reliability, as was done in Dekkers et al. [3]. Instead, we first have to translate $r_{D}^{2}$ into an FI that refers to the full genetic effect, after which we can add this FI to $\theta_{A}$ and finally find the full reliability from Eq. (4). To accomplish this, we translate the reliability of predictions based on deviations of genomic relationships from pedigree relationships, $r_{D}^{2}$, into an FI that refers to the full genetic effect by solving Eq. (4) for $\theta$, resulting in:

9 $$\theta_{{D_{G} }} = \frac{{r_{D}^{2} \left( {1 - r_{D}^{2} h^{2} } \right)}}{{1 - r_{D}^{2} }}.$$

We use the subscript $D_{G}$ here to distinguish $\theta_{{D_{G} }}$, which represents the FI for $g_{G}$ that originates from deviations of genomic from pedigree relationships, from $\theta_{M}$ and from the $\theta_{D}$ given in Dekkers et al. [3], which represent FI for $g_{M}$. In other words, the $\theta_{{D_{G} }}$ in Eq. (9) represents the FI due to genomic relationships deviated from pedigree relationships for estimation of $g_{G}$, rather than $g_{M}$. Parameter $\theta_{{D_{G} }}$ can be solved for by entering $r_{D}^{2}$ into Eq. (9), where $r_{D}^{2}$ is calculated from Eqs. (2b) and (3b). Unfortunately, substitution of Eqs. (2b) and (3b) into Eq. (9) yields a very complex expression and is, therefore, not shown. (Note that Eq. (9) follows from the general Eq. (4), so it is not limited to marker information but can be applied for any source of information). Next, we can compute the total FI for $g_{G}$ as:

$$\theta_{G} = \theta_{A} + \theta_{{D_{G} }} .$$

Finally, the reliability of the total GEBV follows from substituting the resulting $\theta_{G}$ into Eq. (4).

We use a numerical example to illustrate that this approach yields the same result as the SIT-based prediction (Eq. (8)) if we ignore a potential reduction in residual variance due to the merger of pedigree and marker information.

#### Example of the equivalence of the FI and SIT approaches when merging pedigree and genomic information

Suppose $h^{2}$ = 0.3, $M_{e}$ = 400, $N$ = 5000, and $q^{2}$ = 0.8. From Eq. (2b), we find $\theta_{D}$ = 3.0000. From Eq. (3b), using $\theta_{M} = \theta_{D}$, we find $r_{D}^{2}$ = 0.6297. Suppose we have a pedigree-based EBV with the same reliability, $r_{A}^{2}$ = 0.6297. We choose this same value on purpose, so we can easily ignore the reduction in residual variance in the FI approach (i.e., we have a single value for the initial accuracy, which can be used directly in Eq. (4), as explained in the following). First, using SIT, the reliability of the total GEBV follows from Eq. (8), giving $r_{G}^{2}$ = 0.7728. Second, using the FI approach, the FI based on pedigree follows from Eq. (9), using $r^{2}$ = $r_{A}^{2}$ = 0.6297, giving $\theta_{A}$ = 1.3795. Analogously we find $\theta_{{D_{G} }}$ = 1.3795. Note that $\theta_{{D_{G} }}$ is smaller than $\theta_{D}$, because the markers provide less information on $g_{G}$ than on $g_{M}$. The prediction of reliability of the total GEBV based on the FI approach then follows from Eq. (4), using $\theta = \theta_{G} = \theta_{A} + \theta_{{D_{G} }}$ = 2.7590 and $r^{2} = 0.6297$. This yields $r_{G}^{2}$ = 0.7728, which is the same result as obtained with the SIT approach, and illustrates that the SIT and FI approaches yield the same result when the same assumptions are made.

The use of the original $r^{2}$ (i.e*.* 0.6297) in Eq. (4) in the previous paragraph ignores a potential reduction in residual variance due to the merger of pedigree and marker information. Thus, when combining pedigree and genomic information, SIT and FI yield the same accuracy predictions on the condition that: (1) we use a genomic FI that refers to the full genetic effect $g_{G}$, rather than to $g_{M}$, and (2) a potential reduction in residual variance in GP due to the increased amount of information when merging marker and pedigree data is ignored.

A prediction of $r_{G}^{2}$ using the FI approach that accounts for (and assumes) a reduction in residual variance due to the merger of genomic and pedigree information follows from solving Eq. (4) for $r^{2}$, giving:

10 $$r_{G}^{2} = \frac{{1 + \theta_{G} - \sqrt {\left( {1 + \theta_{G} } \right)^{2} - 4h^{2} \theta_{G} } }}{{2h^{2} }}.$$

Using $\theta_{G}$ = 2.7590 in Eq. (10) yields $r_{G}^{2} = 0.7829$. This value is slightly larger than the 0.7728 presented above where we ignored a potential reduction in residual variance when combining pedigree and marker information. However, while it is clear that the residual variance decreases when merging two subpopulations into a single reference population, we are not sure whether this decrease also occurs when merging pedigree and genomic data in a single GP, for example with single step GP [1, 12]. Hence, we draw no conclusions on the superiority of Eq. (10) vs. Eq. (8).

## Conclusions

Existing expressions for the reliability of GEBV can be understood as a proportion of the variance explained. Using this concept, we showed that the apparent discrepancy between predictions of the accuracy of GEBV based the SIT vs. FI approaches in Dekkers et al. [3] originated from two sources. First, the FI in Dekkers et al. [3] referred to the genetic component that is captured by markers, rather than the full genetic component. Second, the SIT approach did not account for the increase in accuracy of GEBV due to a reduction of the residual variance when combining information sources. Our results show that the SIT and FI approaches for combining information for GP are equivalent and provide identical accuracies when the underlying assumptions are equivalent.

## Data Availability

Not applicable.

## Appendices

### Appendix 1

#### Derivation of Eq. 3a for the reliability of GEBV when accounting for the reduction in residual variance based on selection index theory

The accuracy of the estimated effect of one effective segment, accounting for simultaneous fitting of all segments, can be found from selection index theory by including the estimated effects of the other $M_{e} - 1$ segments as an information source. In this way, we account for the reduction in residual variance due to the fitting of the other segments. This approach is an analogy of a pseudo-BLUP selection index, where the EBV of the mates of an individual’s parents are included as an information source [13]. The index to predict the effect of the focal segment then contains two information sources; (1) the mean phenotype of the reference population given the effect of the focal segment, and (2) the contribution of the estimated effects of the other $M_{e} - 1$ segments to the mean phenotype of the reference population. Drawing parallels to estimation of the breeding value of a sire based on the mean of its progeny using pseudo-BLUP to facilitate interpretation of these two information sources, the first information source is analogous to the mean phenotype of the progeny, while the second information source is analogous to the mean EBV of the dams of the progeny. Inclusion of the mean EBV of the dams of the progeny yields a more accurate prediction of the EBV of the sire. Analogously, for genomic prediction (GP), inclusion of the estimated effects of the other $M_{e} - 1$ segments yields a more accurate prediction for the focal segment.

By definition, the $M_{e}$ effective segments are independent (i.e., in linkage equilibrium) and each segment explains an amount $\frac{{h^{2} }}{{M_{e} }}$ of the phenotypic variance. The index weights follow from selection index theory [7] as:

$$\begin{aligned} \left[ {\begin{array}{*{20}c} {b_{1} } \\ {b_{2} } \\ \end{array} } \right] & = {\mathbf{b}} = {\mathbf{P}}^{ - 1} {\mathbf{g}} = \left[ {\begin{array}{*{20}c} {\frac{{q^{2} h^{2} }}{{M_{e} }} + \frac{{1 - \frac{{q^{2} h^{2} }}{{M_{e} }}}}{N}} & {\frac{{r^{2} h^{2} \left( {M_{e} - 1} \right)}}{{M_{e} N}}} \\ {\frac{{r^{2} h^{2} \left( {M_{e} - 1} \right)}}{{M_{e} N}}} & {\frac{{r^{2} h^{2} \left( {M_{e} - 1} \right)}}{{M_{e} N}}} \\ \end{array} } \right]^{ - 1} \left[ {\begin{array}{*{20}c} {q^{2} h^{2} /M_{e} } \\ 0 \\ \end{array} } \right] \\ & = \frac{N}{{Nq^{2} h^{2} + M_{e} - q^{2} h^{2} - r^{2} h^{2} \left( {M_{e} - 1} \right)}} \left[ {\begin{array}{*{20}c} {q^{2} h^{2} } \\ { - q^{2} h^{2} } \\ \end{array} } \right] \\ \end{aligned}$$

The reliability of the resulting prediction can be derived as:

$$\begin{aligned} r^{2} & = \frac{{{\mathbf{b^{\prime}g}}}}{{h^{2} /M_{e} }} = \frac{1}{{h^{2} /M_{e} }} \frac{N}{{Nq^{2} h^{2} + M_{e} - q^{2} h^{2} - r^{2} h^{2} \left( {M_{e} - 1} \right)}} \left[ {\begin{array}{*{20}c} {q^{2} h^{2} } & { - q^{2} h^{2} } \\ \end{array} } \right]\left[ {\begin{array}{*{20}c} {q^{2} h^{2} /M_{e} } \\ 0 \\ \end{array} } \right]. \\ &= q^{2} \frac{{Nq^{2} h^{2} /M_{e} }}{{\frac{{Nq^{2} h^{2} }}{{M_{e} }} + M_{e} - q^{2} h^{2} /M_{e} - r^{2} h^{2} \left( {M_{e} - 1} \right)}} \\ \end{aligned}$$

This result is identical to Eq. (3a), prior to assuming that $h^{2}(q^{2}-r^{2})/M_{e}\ll 1$.

### Appendix 2

#### Derivation of Eq. (7) for the reliability of GEBV when combining subpopulations without accounting for the additional reduction in residual variance

Substituting $\theta_{1}$ and $\theta_{2}$ in the expression for $r^{2}$ by Eq. (6) yields:

$$r^{2} = q^{2} \frac{{\frac{{r_{1}^{2} \left( {1 - \frac{{q^{2} h^{2} }}{{M_{E} }}} \right)}}{{q^{2} - r_{1}^{2} }} + \frac{{r_{2}^{2} \left( {1 - \frac{{q^{2} h^{2} }}{{M_{E} }}} \right)}}{{q^{2} - r_{2}^{2} }}}}{{\frac{{r_{1}^{2} \left( {1 - \frac{{q^{2} h^{2} }}{{M_{E} }}} \right)}}{{q^{2} - r_{1}^{2} }} + \frac{{r_{2}^{2} \left( {1 - \frac{{q^{2} h^{2} }}{{M_{E} }}} \right)}}{{q^{2} - r_{2}^{2} }} + 1 - q^{2} h^{2} /M_{E} }}.$$

Dividing the numerator and denominator by $1 - q^{2} h^{2} /M_{E}$ yields:

$$r^{2} = q^{2} \frac{{\frac{{r_{1}^{2} }}{{q^{2} - r_{1}^{2} }} + \frac{{r_{2}^{2} }}{{q^{2} - r_{2}^{2} }}}}{{\frac{{r_{1}^{2} }}{{q^{2} - r_{1}^{2} }} + \frac{{r_{2}^{2} }}{{q^{2} - r_{2}^{2} }} + 1}}.$$

Writing all terms with $\left( {q^{2} - r_{1}^{2} } \right)\left( {q^{2} - r_{2}^{2} } \right)$ as denominator and then cancelling this denominator yields:

$$\begin{aligned} r^{2} =& q^{2} \frac{{r_{1}^{2} \left( {q^{2} - r_{2}^{2} } \right) + r_{2}^{2} \left( {q^{2} - r_{1}^{2} } \right)}}{{r_{1}^{2} \left( {q^{2} - r_{2}^{2} } \right) + r_{2}^{2} \left( {q^{2} - r_{1}^{2} } \right) + \left( {q^{2} - r_{1}^{2} } \right)\left( {q^{2} - r_{2}^{2} } \right)}} \hfill \\ =& q^{2} \frac{{r_{1}^{2} q^{2} + r_{2}^{2} q^{2} - 2r_{1}^{2} r_{2}^{2} }}{{q^{4} - r_{1}^{2} r_{2}^{2} }} \hfill \\ \end{aligned}$$

Dividing the numerator and denominator by $q^{4}$ yields Eq. (7).

### Appendix 3

#### Proof that the SIT approach yields Eq. (7) when combining subpopulations without accounting for the additional reduction in residual variance

The two non-overlapping reference populations yield GEBV $\hat{g}_{1}$ and $\hat{g}_{2}$. In both populations, the markers capture the same proportion of the genome. Hence, not only is the value of *q*^2^ the same for the two subpopulations, but the markers are also assumed to be associated with the same part of the genome in the two subpopulations. The derivation uses $\sigma_{g}^{2} = 1$, such that $\sigma_{{g_{M} }}^{2} = q^{2}$, which is the variance of the (true) genetic component captured by markers. The index for the combined GEBV is:

$$\hat{g} = b_{1} \hat{g}_{1} + b_{2} \hat{g}_{2} .$$

The index weights follow from:

$${\mathbf{b}} = {\mathbf{P}}^{ - 1} {\mathbf{g}} = \left[ {\begin{array}{*{20}c} {r_{1}^{2} } & {\frac{{r_{1}^{2} r_{2}^{2} }}{{q^{2} }}} \\ {\frac{{r_{1}^{2} r_{2}^{2} }}{{q^{2} }}} & {r_{2}^{2} } \\ \end{array} } \right]^{ - 1} \left[ {\begin{array}{*{20}c} {r_{1}^{2} } \\ {r_{2}^{2} } \\ \end{array} } \right] = \frac{1}{{1 - r_{1}^{2} r_{2}^{2} /q^{4} }}\left[ {\begin{array}{*{20}c} {1 - r_{2}^{2} /q^{2} } \\ {1 - r_{1}^{2} /q^{2} } \\ \end{array} } \right].$$

The off-diagonal element of the variance matrix, $\frac{{r_{1}^{2} r_{2}^{2} }}{{q^{2} }}$, follows from Fig. 2 of [14], and deviates from the intuitively expected value of $r_{1}^{2} r_{2}^{2}$ because $\hat{g}_{1}$ and $\hat{g}_{2}$ capture the same marker-associated part of the genome, such that $cov\left( {\hat{g}_{1} ,\hat{g}_{2} } \right)$ = $r_{M,1}^{2} r_{M,2}^{2} \sigma_{{g_{M} }}^{2}$ = $\left[ {r_{1}^{2} /q^{2} } \right] \left[ {r_{2}^{2} /q^{2} } \right] q^{2}$ = $r_{1}^{2} r_{2}^{2} /q^{2}$.

The reliability of the resulting combined GEBV is:

$$r^{2} = \frac{{{\mathbf{b^{\prime}g}}}}{{\sigma_{g}^{2} }} = \frac{1}{{1 - r_{1}^{2} r_{2}^{2} /q^{4} }}\left[ {\begin{array}{*{20}c} {1 - \frac{{r_{2}^{2} }}{{q^{2} }}} \\ {1 - \frac{{r_{1}^{2} }}{{q^{2} }}} \\ \end{array} } \right]^{^{\prime}} \left[ {\begin{array}{*{20}c} {r_{1}^{2} } \\ {r_{2}^{2} } \\ \end{array} } \right] = \frac{{r_{1}^{2} + r_{2}^{2} - 2r_{1}^{2} r_{2}^{2} /q^{2} }}{{1 - r_{1}^{2} r_{2}^{2} /q^{4} }},$$

which is identical to Eq. (7).
